# Serum extracellular vesicle MicroRNAs as candidate biomarkers for acute rejection in patients subjected to liver transplant

**DOI:** 10.3389/fgene.2022.1015049

**Published:** 2022-10-13

**Authors:** Wenjing Wang, Wen Li, Li Cao, Bo Wang, Chang Liu, Yannan Qin, Bo Guo, Chen Huang

**Affiliations:** ^1^ Department of Surgical Intensive Care Unit, The First Affiliated Hospital of Xi’an Jiaotong University, Xi’an, China; ^2^ Department of Cell Biology and Genetics, School of Basic Medical Sciences, Xi’an Jiaotong University Health Science Center, Xi’an, China; ^3^ Institute of Genetics and Developmental Biology, Translational Medicine Institute, School of Basic Medical Sciences, Xi’an Jiaotong University Health Science Center, Xi’an, China; ^4^ Department of Hepatobiliary Surgery, The First Affiliated Hospital of Xi’an Jiaotong University, Xi’an, China

**Keywords:** acute rejection, liver transplantaion, biomarker, extracellular vesicle, microRNA

## Abstract

Acute rejection (AR) is a common and grave complication of liver transplantation (LT). The diagnosis of AR is challenging because it has nonspecific clinical features and requires invasive procedures. Since extracellular vesicles (EVs) are promising candidates as indicators for diagnosis of various diseases, this study aimed to identify serum EV microRNAs (miRNAs) as potential biomarkers for AR in patients subjected to LT. We collected clinical information and serum samples from the liver transplant recipients with and without AR (non-AR). EVs from the serum were isolated *via* ultracentrifugation and identified using transmission electron microscopy, nanoparticle tracking analysis, and western blotting. EV RNA was extracted and sequenced on an Illumina HiSeq 2500/2000 platform to identify differentially expressed miRNAs between the groups. Gene Ontology (GO) and Kyoto Encyclopedia of Genes and Genomes (KEGG) enrichment analyses were performed on the target gene candidates of the differentially expressed miRNAs to test their functions in biological systems. Then, we validated 12 differentially expressed miRNAs by quantitative real-time PCR. The results demonstrated that 614 EV miRNAs were significantly altered (387 up regulated and 227 down regulated) between non-AR and AR patients. GO enrichment analysis revealed that these target genes were related to cellular processes, single-organism processes, biological regulation, metabolic processes, cells, cell parts, protein-binding processes, nucleoid binding, and catalytic activity. Furthermore, KEGG pathway analysis demonstrated that the target genes of the differentially expressed miRNAs were primarily involved in ubiquitin-mediated proteolysis, lysosomes, and protein processing in the endoplasmic reticulum. miR-223 and let-7e-5p in AR patients were significantly up-regulated compared to those in non-AR patients, whereas miR-199a-3p was significantly down-regulated, which was consistent with sequencing results. The expression of serum EV miRNAs (up-regulated: miR-223 and let-7e-5p and miR-486-3p; down regulated: miR-199a-3p, miR-148a-3p and miR-152-3p) in AR patients was significantly different from that in non-AR patients, and these miRNAs can serve as promising diagnostic biomarkers for AR in patients subjected to liver transplant.

## Introduction

Liver transplantation (LT) has become the most effective curative therapy for end-stage liver disease with an irreversible degradation of liver function. However, serious postoperative complications remain a major challenge limiting the long-term success of LT (Meirelles Junior et al., 2015). Rejection is an immune reaction in which a recipient lymphatic system recognizes and activates graft-specific antigens and attacks and damages grafts. Acute rejection (AR) is one of the most common and intractable complications of graft introduction, with the one-year incidence rate of 11.5% (age dependent range 9.4%–20.5%) (Kim et al., 2016; Schütz et al., 2017). It usually occurs within the first 3 months and affects the function of the transplanted liver and the prognosis of patients. Traditional methods are affected by many factors that lack specificity and sensitivity. The gold standard for diagnosing AR is percutaneous liver biopsy; however, it still faces the challenge of being invasive and delayed. The accuracy of pathological diagnosis is affected by factors, such as the experience level of the observing pathologists and the sampling quality (Jadlowiec and Taner, 2016; Rastogi, 2022). The onset of symptoms marks the transition from subclinical to clinical disease, and genetic changes occur before the onset of clinical symptoms. Therefore, a method to non-invasively identify early biomarkers at the cellular and molecular levels is urgently needed to diagnose AR (Krenzien et al., 2019).

Extracellular vesicles (EVs) are nano-sized membrane vesicles carrying proteins, mRNAs, and other small RNAs found in most biological fluids (Thery, 2011). They are released into the extracellular region by most cell types and act as mediators in cell-cell communication, modulating various types of physiological processes, including immune disorders (Obregon et al., 2006; Valadi et al., 2007; McLellan, 2009; van der Vlist et al., 2012). They have also shown great potential as diagnostic or therapeutic tools (Lin et al., 2015). Some studies also highlighted the role of EVs in the alternation of the immune response in the field of transplant immunology. T cells release EV carrying miR-142-3p, which are transferred to endothelial cells and impair endothelial integrity *via* down-regulation of RAB11FIP2 during cardiac allograft rejection (Dewi et al., 2017). Another study using high-throughput sequencing of the EV microRNA (miRNA) profile in the peripheral blood revealed 52 known and 5 conserved EV miRNAs as diagnostic biomarkers specifically expressed in recipients with delayed graft function (Wang et al., 2019).

miRNAs play important roles in numerous cellular functions by mediating gene expression post-transcriptionally. Recent studies have shown that miRNAs showed different expression profiles in patients subjected to organ transplantation (Afshari et al., 2021; Boštjančič et al., 2021). Increasing publications suggests that miRNAs may serve as potential non-invasive biomarkers for AR during transplant (Hamdorf et al., 2017; Lin et al., 2021). miRNAs could be detected in nearly all body fluids (serum, bile, and urine) and have been proposed to be potential biomarkers for diagnosis and therapy (Cortez et al., 2011). EV miRNAs may be more stable and sensitive than circulating free miRNAs. However, to our knowledge, hardly any studies have reported EV miRNAs as biomarkers for AR after LT. Hence, the present study investigated the hypothesis that differentially expressed immune-related EV miRNAs may provide key information about allograft status and can be used as circulating markers for diagnosing AR after LT. This study also identified functions and signaling pathways of the differentially expressed EV miRNA target genes. The findings of this study contribute to the investigation of the feasibility of EV miRNAs as liquid biopsy biomarkers for patients with AR after LT.

## Materials and methods

### Patients and samples

Sixty patients who underwent LT at the First Affiliated Hospital of Xi’an Jiaotong University between 2016 and 2019 were included in this study (Supplementary Table S1). Thirty patients were diagnosed with T-cell mediated AR *via* liver biopsy using the Banff criteria based on clinical suspicion. Another 30 transplant patients without AR (non-AR) after surgery were considered as controls and followed up regularly every 2–4 weeks. Every patient received a graft *via* donation after a citizen’s death. Informed consent was obtained from all patients or their legally authorized representatives. This study was approved by the Ethics Committee of Xi’an Jiaotong University. Serum samples of patients with AR were collected immediately after onset, and then, the initial treatment was carried out. Non-AR sera were obtained on the date matching patients with AR as much as possible (7–55 days after LT). Serum samples were divided into a test set (*n* = 10 each for AR and non-AR) and a verification set (*n* = 20 each for AR and non-AR). All serum samples were stored at −80°C until use.

### Extracellular vesicle isolation

EVs were isolated using an ExoQuick™ Exosome Precipitation Solution (System Biosciences, United States) according to the manufacturer’s instructions. First, the serum was centrifuged at 2000 × *g* for 15 min to remove the cells and cell debris. The supernatant obtained was diluted in 500 μl Dulbecco’s phosphate-buffered saline (DPBS) at equal volumes and then mixed with 252 μl ExoQuick exosome precipitation solution. After incubation at 4°C for 1 h, the obtained mixture was centrifuged at 1,500 × *g* for 0.5 h. Finally, the supernatant was discarded and EVs pellets were diluted in 150 μl DPBS.

### Extracellular vesicle identification

The EV suspension (10 μl) was loaded onto a copper grid and was stained with uranyl acetate for 1 min. The grids were washed and dried with filter paper for 10 min at room temperature, and the morphology of the EVs was observed under a transmission electron microscope (Hitachi, Japan) at a voltage of 100 kV. The average concentration and size distribution of EVs were determined using nanoparticle tracking analysis (NanoFCM, China).

Dilutions of the EV suspension were also prepared. Briefly, 10 μl of the original EV suspensions were diluted at 1/10, 1/100, and 1/1,000. Anti-mouse/rat CD81 antibodies fluorescently labeled with PE (BioLegend, United States) and APC-conjugated anti-mouse CD63 antibodies (BioLegend, United States) were incubated with 100 μl of the diluted EVs suspensions for 15 min at room temperature in the dark, and the proportion of CD 81 and CD 63 positive cells was determined using a flow cytometer (Beckman, United States).

### RNA extraction from extracellular vesicle

RNA was extracted from the EV pellets using an Exosomal RNA Isolation Kit (Norgen, United States), according to the manufacturer’s protocol. RNA concentration was measured using Nanodrop 2000 (Thermo Fisher Scientific, United States).

### RNA sequencing

Three micrograms of total RNA was used for the construction of a small RNA library by a NEBNext^®^ Multiplex Small RNA Library Prep Set for Illumina^®^ (NEB, United States) according to the manufacturer’s instructions. Index codes were added to the attribute sequences of each sample. Agilent Bioanalyzer 2100 system with DNA High Sensitivity Chips was used for quality check of the library. Clustering of the index-coded samples was performed on a cBot Cluster Generation System using a TruSeq SR Cluster Kit v3-cBot-HS (Illumina, United States) according to the manufacturer’s instructions. After cluster generation, the library preparations were sequenced on an Illumina HiSeq 2500/2000 platform and 50-bp single-end reads were generated.

### Data processing and bioinformatics analysis

Briefly, clean reads were obtained from raw data by removing reads containing adapter dimers, junk, and low-quality reads, and a certain range of lengths from clean reads was chosen for further analyses. The small RNA tags were mapped to search for known miRNAs using miRBase 20.0 as reference. Custom scripts were used to obtain the miRNA counts and base bias at the first position of the identified miRNA with a certain length and at each position of all identified miRNAs, respectively. Novel miRNAs were predicted using two software packages (miREvo and mirdeep2) by exploring their secondary structures. Differential miRNA expression analysis was performed using the DEGseq (2010) R package. A q value <0.01 and log_2_ (fold change) > 1 were set by default as the threshold for significant differential expression. To predict the genes targeted by the most abundant miRNAs, computational target prediction algorithms (miRanda 3.3a) were used to identify miRNA-binding sites. Finally, Gene Ontology (GO) and Kyoto Encyclopedia of Genes and Genomes (KEGG) enrichment analyses were performed to reveal functions and pathways.

### Quantitative real-time PCR

To validate the sequencing results, qRT-PCR was performed using the SYBR Green PCR kit (GenStar, China), according to the manufacturer’s instructions. Assays were performed on AR and non-AR samples (*n* = 20 each) for 12 immune-related miRNAs that met the defined criteria. Each reaction was performed in a 20 μl volume containing 2 μl cDNA, 1 μl each primer, 10 μl SYBR Green, and 6 μl RNase-free water. All reactions were performed in triplicate using an IQ5 Multicolor Real-Time PCR Detection System (Bio-Rad, United States). The 2^−ΔΔCt^ method was used for quantitation and U6 was used as the miRNA control. The Ct value range of qRT-PCR is from 14 to 32, and fold change <0.5 or >2 was considered to be significantly down-regulated or up-regulated, respectively. The primer sequences used are listed in Supplementary Table S2.

### Statistical analysis

All experiments were performed in triplicate and statistical analyses were performeded using GraphPad Prism (San Diego, United States). For miRNA expression, Student’s t-test was used to analyze the differences between two independent groups. Data were reported as the mean ± SD, and *p*-values < 0.05 was considered statistically significant.

## Results

### Clinical information

**FIGURE 1:**
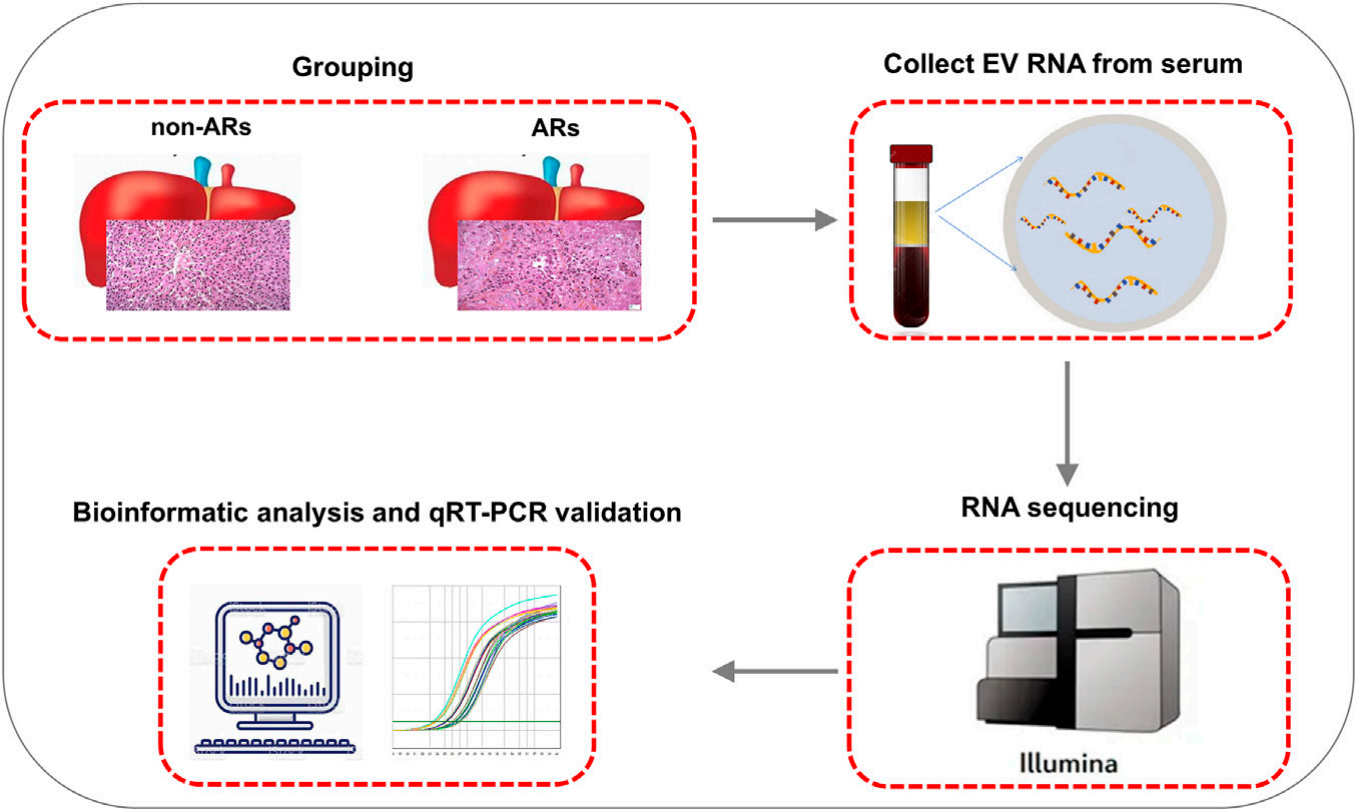
Schematic illustration of the workflow: From blood collection to analysis and validation.

Immunosuppressive treatment consists of tacrolimus/cyclosporine, mycophenolate mofetil, and prednisone was administered to recipients after transplantation to prevent AR. Basic information regarding the liver transplant recipients is shown in Supplementary Table S1. We found that the AR and non-AR patients did not differ significantly in terms of sex, etiology, Child-Pugh score, model for end-stage liver disease scores (MELD scores), cold/warm ischemia time, and ABO compatibility. However, the mean immunosuppressive concentration in AR patients during the first week of treatment was significantly lower than non-AR patients (*p* < 0.05) (Supplementary Table S1). Selected serum samples were then collected for EV isolation and sequencing analysis to screen potential biomarkers for the diagnosis of AR (Figure 1).

### Extracellular vesicle isolation and validation

**FIGURE 2:**
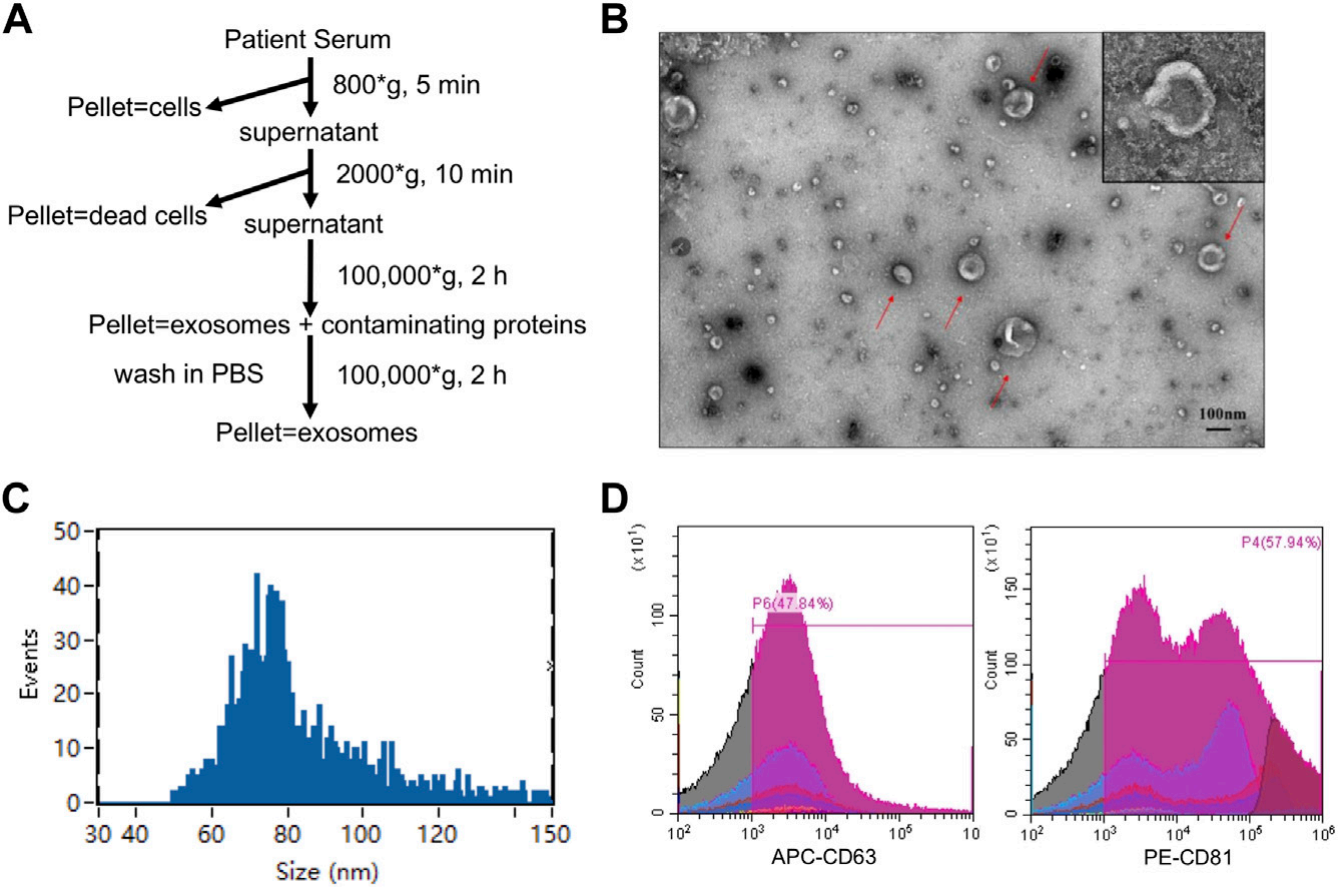
Characterization of EVs. **(A)**The procedures for EVs isolation used in this study. **(B)** Morphological characterization of EVs isolated from serum by transmission electron microscopy. Typical EVs were amplified with red arrows. Scale bar = 100 nm (upper). **(C)** Average size distribution of EVs was revealed by nanoparticle tracking analysis. **(D)** EVs protein markers (CD 63 and CD 81) were identified by flow cytometry.

We isolated EV from the serum of all participants by ultracentrifugation as previously reported (Thery et al., 2006) (Figure 2A). Transmission electron microscopy revealed the morphology of the EV isolated from patient serum (Figure 2B), which was consistent with previous reports. Nanoparticle tracking analysis revealed an average diameter of approximately 85.52 nm (Figure 2C), suggesting sufficient EV purification. Flow cytometry was also performed to detect two commonly used EV protein markers: CD63 and CD81. The results showed that both protein markers were highly expressed in the isolated EV (Figure 2D). Taken together, we confirmed the successful purification of EV from all serum samples.

### Small RNA sequencing analysis

**FIGURE 3:**
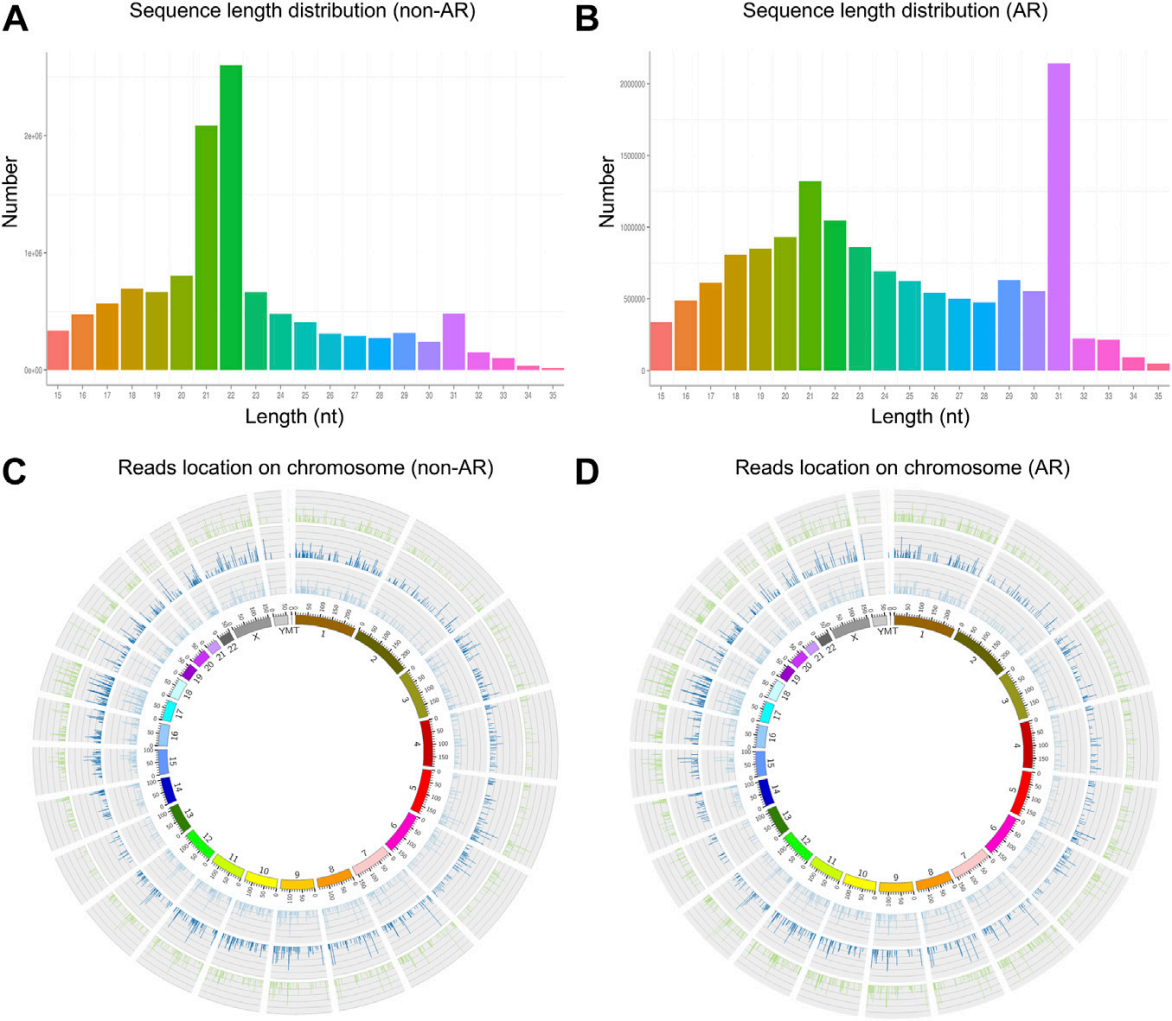
The length distribution of the clean reads sequence and location of clean reads on each chromosome. **(A,C)** non-AR patients. **(B,D)** AR patients.

We obtained 13,067,031 and 15,270,653 total reads from non-AR and AR patients with solexa high-throughput sequencing. After removing the reads containing poly-N, length <15 or >35, and low-quality reads, 11,982,753 (91.7%) and 13,990,228 (91.6%) high-quality clean reads were extracted from the two groups, respectively (Table 1). The distributions of sequence length were then analyzed, and a certain length range was selected. The lengths of the clean reads peaked at 21–22 nt, which are within the range for miRNAs (Figures 3A,B). Approximately 62% and 18.78% of sRNA could be mapped to the genome in non-AR and AR patients using Bowtie software (Langmead et al., 2009) (Table 2). In addition, density statistics of the reads of each chromosome on the genome of each sample were determined, and the distribution of the reads on each chromosome was checked using Circos mapping (Figures 3C,D). Next, the reads of rRNA, tRNA, snoRNA, and other snRNAs were annotated and removed for subsequent analysis (Supplementary Figure S1; Supplementary Table S3).

**TABLE 1:** Raw data quality control.

Sample	Total reads	Containing “N” reads	Low quality	Length<15	Length>35	Clean reads
non-AR	13,067,031	0	134	821,015	263,129	11,982,753
AR	15,270,653	0	107	858,714	421,604	13,990,228

**TABLE 2:** Reads mapping to the reference sequence.

Sample	Total reads	Mapped reads	Mapped reads (+)	Mapped reads (−)
non-AR	6,934,287	4,299,581	2,134,476	2,165,105
AR	5,868,860	1,102,076	849,059	253,017

### Differentially expressed miRNAs

**FIGURE 4:**
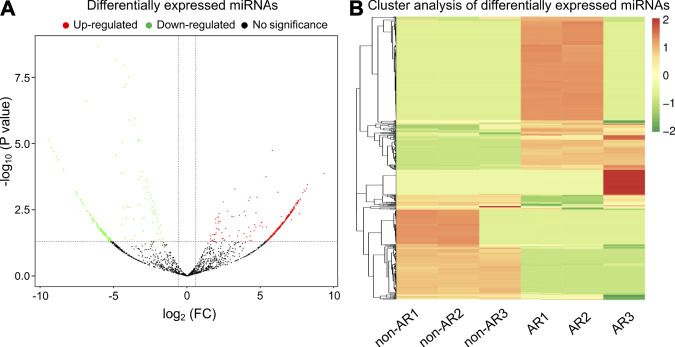
Volcano plot and heat map of differential expressed miRNAs obtained from the comparison of non-AR and AR patients. **(A)** Each point in the volcano plot represents one miRNA. Red points represent upregulated ones, green points represent downregulated ones, and dark points represent no significant changes in miRNAs. **(B)** The abscissa represents different groups, the ordinate represents the different miRNAs compared in the groups, and the color blocks at different positions represent the relative miRNA expressions.

Mapped small RNA tags and miRBase 20.0 were used to identify known miRNAs. A total of 515 and 397 mature miRNAs were identified, respectively (Table 3). miRNA expression levels were estimated as transcript per million (TPM) and differential expression analysis was performed between the non-AR and AR patients (Supplementary Figure S2; Supplementary Table S4; Supplementary Table S5). Compared to the non-AR patients, 387 miRNAs (including miR-96-5p, miR-1290, miR-19b-3p, and miR-30b-5p) were significantly upregulated, whereas 227 miRNAs (including miR-134-5p, miR-455-5p, miR-10a-5p, and miR-194-5p) were downregulated in patients with AR (Figures 4A,B).

**TABLE 3:** Known miRNAs obtained.

Sample	Mapped Known-miRNAs	Mapped Novel-miRNAs	Mapped total miRNAs
non-AR	515	862	1,377
AR	397	554	951

### Gene ontology analysis of the candidate target genes of differentially expressed miRNAs

**FIGURE 5:**
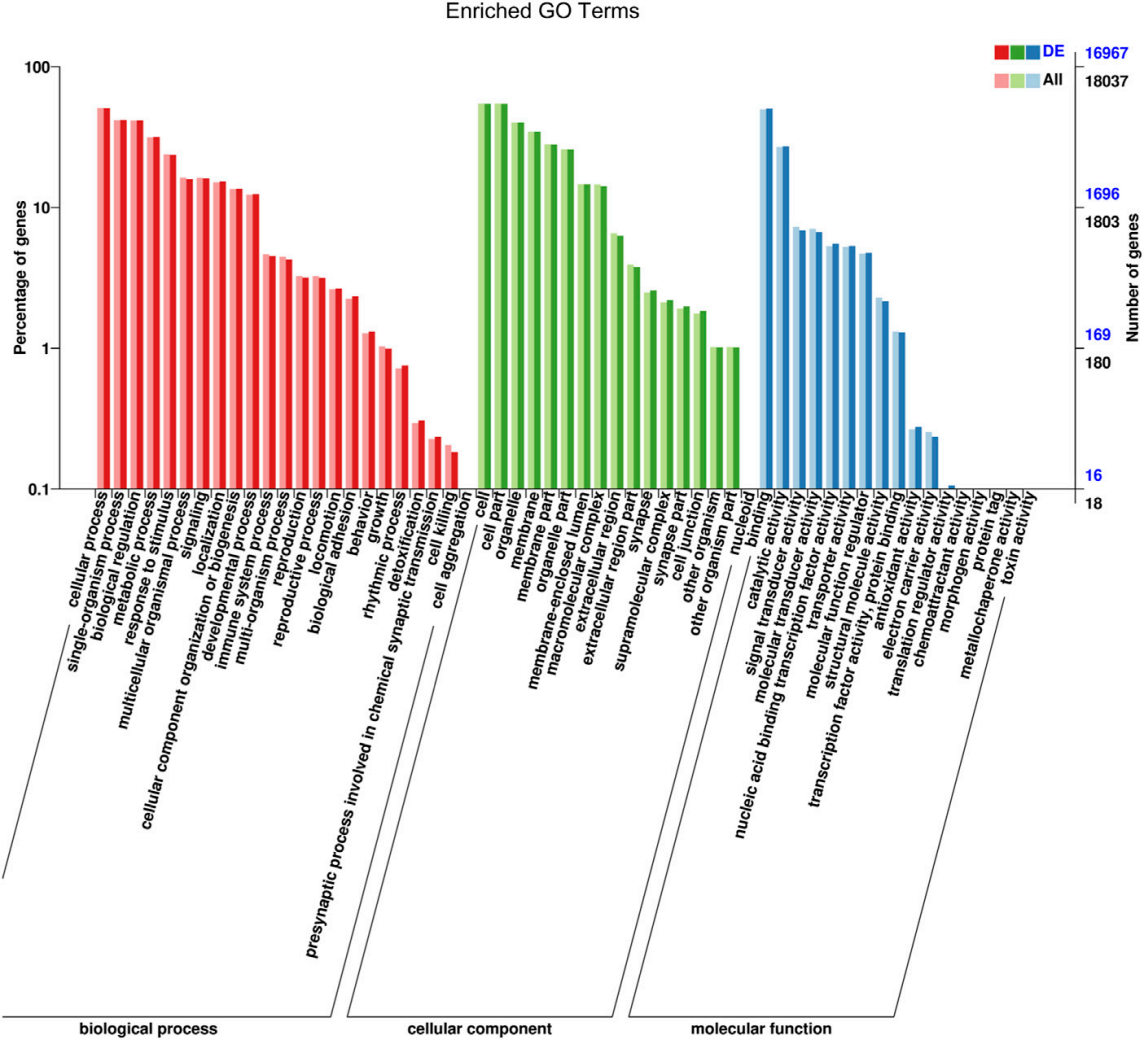
GO function classification target genes of known differential expressed miRNAs in AR patients compared with non-AR patients. Abscissa: class classification. The three different classifications represent the three basic classifications of GO terms. From left to right they are biological process, cellular component, and molecular function.

Target genes of miRNAs were predicted using RNAhybrid and miRanda. The distribution of candidate genes of differentially expressed miRNAs in GO was compared with that of the references, and the number of genes of the significantly enriched GO terms was counted to determine the biological functions that were significantly correlated (Supplementary Table S6). The enrichment results showed that the target genes were related to cellular processes, single-organism processes, biological regulation, metabolic processes, cells, cell parts, protein binding processes, nucleoid binding, and catalytic activity (Figure 5; Supplementary Figure S3).

### Kyoto encyclopedia of genes and genomes pathway analysis of the candidate target genes of the differentially expressed miRNAs

**FIGURE 6:**
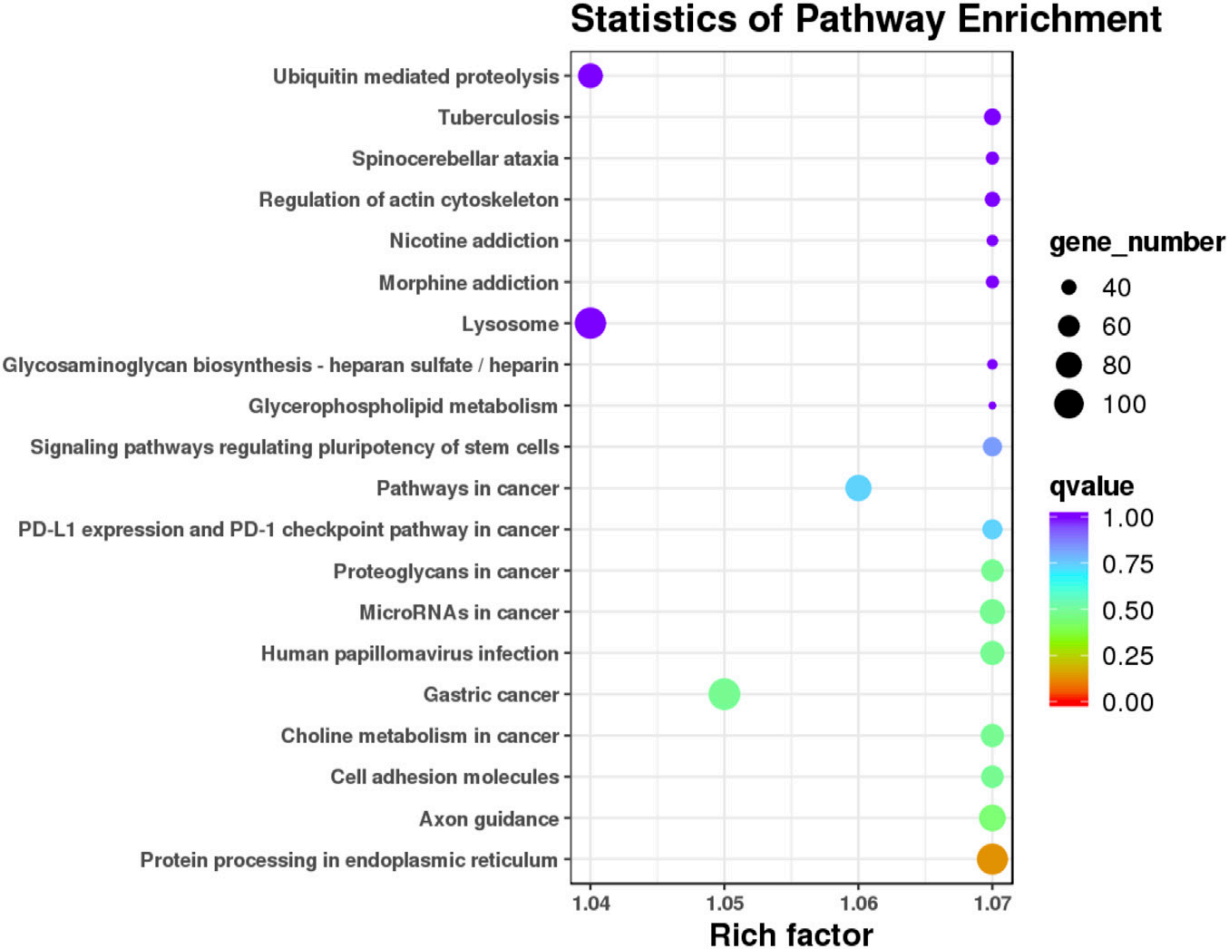
KEGG pathway analysis of the target genes of known differential expressed miRNAs in AR patients compared with non-AR patients. Horizontal axis: Rich factor. The larger the point, the higher the enrichment degree, the more candidate target genes in this pathway, and the color of the point correspond to a different *q* value range. Vertical axis: The definition of the pathway.

The target candidate genes were mapped to reference pathways recorded in the KEGG database to identify the biological pathways through which the differentially expressed miRNAs were involved in AR (Supplementary Table S7). KEGG pathway analysis revealed 20 major pathways occupied by the most abundant target genes of differentially expressed miRNAs. In patients with AR, the target gene enrichment pathways included ubiquitin-mediated proteolysis, lysosomes, and protein processing in the endoplasmic reticulum (Figure 6). These results revealed the potential function of miRNA targets, which may form a regulatory network and play a vital role in the disease process of AR.

### Validation of the potential extracellular vesicle miRNA biomarkers for acute rejection

**FIGURE 7:**
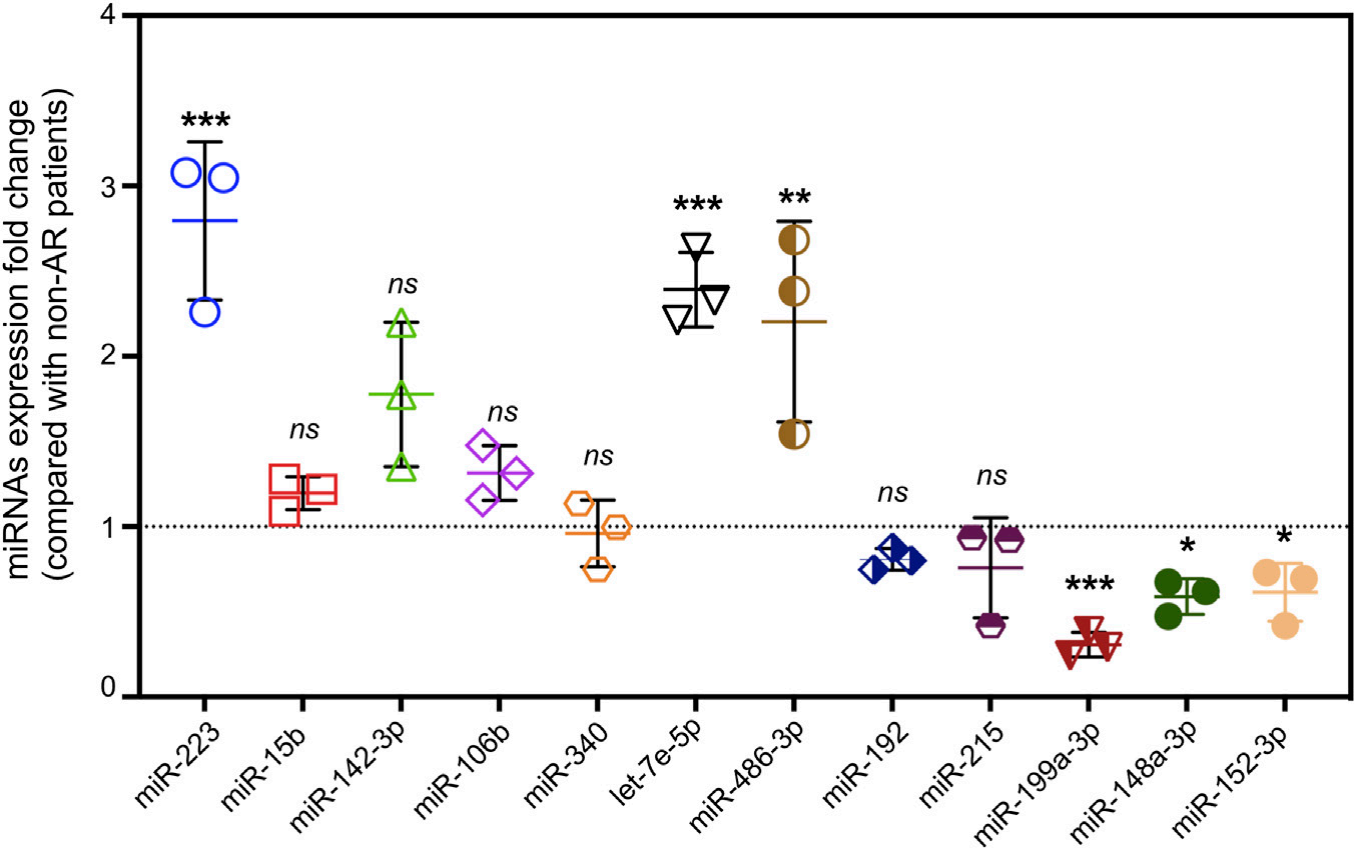
Validation of differential expressed miRNAs by qRT-PCR. miRNA expression of fold change detected by qRT-PCR in AR patients compared with non-AR patients (*n* = 3, p* < 0.05, p** <0.01, p*** <0.001, *ns* = no significance).

To narrow down the potential candidates, miRNAs were screened from immune-related literatures published on PubMed first. Then, 12 hsa-miRNAs with a significant and consistent fold-change were selected for further validation by RT-qPCR. Notably, three of these miRNAs (miR-223, let-7e-5p, and miR-486-3p) were up regulated, whereas miR-199a-3p, miR-148a-3p, and miR-152-3p were significantly down regulated, exhibiting a similar trend from the RNA sequencing data (Figure 7). We then analyzed the correlation between these differentially expressed miRNAs and clinical information, and found that miR-223 was positively correlated with lymphocytes in patients with AR (Table 4).

**TABLE 4:** Correlation Analysis Between differentially expressed EV miRNAs and Clinical Information.

Parameter	miR-223		let-7e		miR-199a-3p	
	*r*	*p* value	*r*	*p* Value	*r*	*p* value
EO (10^9^/L)	0.023	0.950	0.392	0.263	0.226	0.626
LYM (10^9^/L)	0.709	0.022*	0.510	0.132	−0.176	0.277
ALT (U/L)	0.097	0.079	0.499	0.142	−0.731	0.269
AST (U/L)	0.411	0.238	0.035	0.924	−0.546	0.066
GGT (U/L)	0.311	0.382	0.243	0.498	−0.129	0.688
ALP (U/L)	0.178	0.623	0.495	0.176	−0.210	0.194
TBIL(μmol/L)	0.231	0.151	0.176	0.278	−0.048	0.768

## Discussion

AR is a complex immunological process mediated by a variety of cells (including lymphocytes, dendritic cells, and macrophages) and antibodies (Charlton et al., 2018). The main mechanism for AR may be defined as when the recipient lymphatic system recognizes and activates graft-specific antigens, attacks and damages grafts, while humoral immunity gradually develops (Dogan et al., 2018). Most biomarkers are proinflammatory factors, immunomodulatory cytokines, and inflammation-related proteins that could overlap across diseases (Naesens and Anglicheau, 2018; Mirzakhani et al., 2019; Kohut et al., 2020). For example, IL-2 promotes proliferation and differentiation of T cells and its subsets, stimulates the maturation of B cells, and mediates graft rejection (Holzknecht and Platt, 2000). Cytotoxic T lymphocytes act as effector cells that mediate graft rejection. The CD4/CD8 ratio can be used to monitor immune function since a low CD4/CD8 ratio in graft tissues or serum is associated with AR (Shenoy et al., 2012). Many clinical trials have shown that intracellular ATP levels in CD4^+^ T cells can act as markers for identifying infection or allograft rejection (Israeli et al., 2008). However, iATP seems to be more effective in identifying over-immunosuppressed patients with infection than rejection, and should be used in combination with other biomarkers to improve the efficacy of diagnosis (Millan et al., 2009). With the development of technology, some relatively noninvasive and continuous detection methods have been developed, such as cytokines, CD families, and proteomics, among others (Perottino et al., 2022). Lun et al. (2002) and Chae et al. (2018) found that IL-2, IL-6, and IL-17 production by T cells significantly increased during AR. Massoud et al. (2011) previously screened and found 41 highly expressed proteins (such as Hsp60, Hsp70, CC1q, CC3, CC4, CD33, and other immune-activating mediators) in the serum of patients with AR after LT compared to those with non-AR. After verifying independent samples, complement C4 was found to be a good predictor of AR in liver transplant recipients. Besides, many studies found that graft-derived cell-free DNA acted as a non-invasive early rejection and graft damage marker for LT (Schütz et al., 2017; Baumann et al., 2022; Levitsky et al., 2022). However, these studies are still in the experimental research stage; hence their detection efficiency is relatively limited and they have not yet been applied in large-scale clinical practice.

Mounting evidence has proven that miRNAs play critical roles in immune cell development, immune responses, and immune tolerance. As novel diagnostic markers, miRNAs found in EV can be used as noninvasive sensors for acute and chronic transplant rejection (Hamdorf et al., 2017). The significance of this potential value in liver transplant rejection is supported by an animal study conducted by Morita et al. (2014) They investigated the miRNAs involved in the AR of liver allografts in mice and found that levels of miR-146a, -15b, -223, -23a, -27a, -34a, and -451 were significantly increased in the grafts, while those of miR-101a, -101b, and -148a were decreased. A study on human renal transplantation that detected differentially expressed miRNAs found that miR-223 was overexpressed in AR biopsies compared with that in normal allograft biopsies (Ledeganck et al., 2019). Indeed, among the miRNAs overexpressed in AR biopsies, miR-223 levels were the highest; they were also highly expressed in normal human peripheral blood mononuclear cells (PBMCs). Further investigation showed that the activation of PBMCs with the mitogen phytohemagglutinin resulted in a reduction in miR-223 expression (Anglicheau et al., 2009). Let-7, as a highly conserved miRNA family, plays an anti-inflammatory role. Intranasal administration of let-7 mimics reduced IL-13 levels in allergic lungs and alleviated asthma features, such as airway hyper responsiveness, airway inflammation, and goblet cell metaplasia (Kumar et al., 2011). In hematopoietic stem cell transplantation, the study performed by Rachel et al. revealed that miR-199 expression was lower in the EV fraction of patients who subsequently developed acute graft-versus-host disease (aGvHD), verifying the capacity of circulating miR-199 as a diagnostic and prognostic aGvHD biomarker (Crossland et al., 2016). To our knowledge, for the first time in patients subjected to LT, the present study showed a significant elevation in miR-223 and let-7e-5p levels and a reduction of miR-199a-3p levels in EV from serum of patients with those in AR compared with non-AR. This was consistent with the results of other studies of immune disorders; hence, we propose that these EV miRNAs might be novel diagnostic biomarkers for AR.

Selecting one or more closely associated miRNAs would be helpful in exploring the role of EV miRNAs in the mechanism of AR. It is well known that miRNAs regulate gene expression by targeting mRNAs and act as master regulators of various signaling pathways. Therefore, the first principle is to select miRNAs that closely match the genetic background of the target diseases. AR has been shown to be closely related to immune disorders. Based on the results of GO enrichment analysis, we selected 12 miRNAs closely related to immunity for further experimental validation, of which 3 (miR-223, let-7e-5p, and miR-199a-3p) were significantly associated with AR. Second, the choice should be based on KEGG enrichment analyses. Our present data showed that ubiquitin-mediated proteolysis, a widespread and important type of protein regulation at the post-translational level is a reliable pathway. Thus, miR-223 and miR-199a should be chosen for further study, as they have been associated with esophageal squamous cell carcinoma (Kurashige et al., 2012) and human end-stage dilated cardiomyopathy (Baumgarten et al., 2013). However, this study had some limitations. First, we did not examine the correlation between the target genes and candidate miRNAs in patients with AR. This may help explain how EV miRNAs affect the expression of target genes and potential signaling pathways, and will be addressed in our future research. Second, the sample size was relatively limited, so further research involving more samples or establishing multicenter collaboration would be beneficial. Moreover, single biomarkers have insufficient sensitivity and specificity. Therefore, multiple biomarker panels, such as patient clinical manifestations, laboratory results, and other biomarkers, could provide a more robust diagnosis of AR in LT. We hope that our results may provide a reliable serum biomarker for diagnosing AR and uncovering its potential molecular mechanisms in the future.

## Conclusion

In conclusion, this study was the first attempt to screen EVs-based biomarkers for patients with AR after LT, and we analyzed the analyzing differential miRNA expression profiles through high-throughput sRNA sequencing and experimental validation. The expression levels of serum EV miRNAs (up-regulated: miR-223 and let-7e-5p and miR-486-3p; down regulated: miR-199a-3p, miR-148a-3p and miR-152-3p) in patients with AR are significantly different from those in patients with non-AR and can serve as promising diagnostic biomarkers for AR in patients subjected to liver transplant.

## Data Availability

All data are available within this manuscript and its Supplementary Material. sRNA-seq data has been uploaded to SRA repository (PRJNA885293).

## Abbreviations

r, Correlation Coefficient; EO, Eosnophils; LYM, Lymphocyte; ALT, Alanine Aminotransferase; AST, Aspartate Aminotransferase; GGT, Gamma-Glutamyl Transferase; ALP, Alkaline Phosphatase; TBIL, Total Bilirubin.

## References

[B1] Afshari A. Yaghobi R. Karimi M. H. Mowla J. (2021). Alterations in MicroRNA gene expression profile in liver transplant patients with hepatocellular carcinoma. BMC Gastroenterol. 21 (1), 262. 10.1186/s12876-020-01596-2 34118888 PMC8199419

[B2] Anglicheau D. Sharma V. K. Ding R. Hummel A. Snopkowski C. Dadhania D. (2009). MicroRNA expression profiles predictive of human renal allograft status. Proc. Natl. Acad. Sci. U. S. A. 106 (13), 5330–5335. 10.1073/pnas.0813121106 19289845 PMC2663998

[B3] Baumann A. K. Beck J. Kirchner T. Hartleben B. Schütz E. Oellerich M. (2022). Elevated fractional donor-derived cell-free DNA during subclinical graft injury after liver transplantation. Liver Transplant. 16. 10.1002/lt.26479 35429207

[B4] Baumgarten A. Bang C. Tschirner A. Engelmann A. Adams V. von Haehling S. (2013). TWIST1 regulates the activity of ubiquitin proteasome system via the miR-199/214 cluster in human end-stage dilated cardiomyopathy. Int. J. Cardiol. 168 (2), 1447–1452. 10.1016/j.ijcard.2012.12.094 23360823

[B5] Boštjančič E. Večerić-Haler Ž. Kojc N. (2021). The role of immune-related miRNAs in the pathology of kidney transplantation. Biomolecules 11 (8), 1198. 10.3390/biom11081198 34439863 PMC8393721

[B6] Chae M. S. Kim J. W. Chung H. S. Park C. S. Lee J. Choi J. H. (2018). The impact of serum cytokines in the development of early allograft dysfunction in living donor liver transplantation. Medicine 97 (16), e0400. 10.1097/MD.0000000000010400 29668595 PMC5916661

[B7] Charlton M. Levitsky J. Aqel B. O'Grady J. Hemibach J. Rinella M. (2018). International liver transplantation society consensus statement on immunosuppression in liver transplant recipients. Transplantation 102 (5), 727–743. 10.1097/Tp.0000000000002147 29485508

[B8] Cortez M. A. Bueso-Ramos C. Ferdin J. Lopez-Berestein G. Sood A. K. Calin G. A. (2011). MicroRNAs in body fluids--the mix of hormones and biomarkers. Nat. Rev. Clin. Oncol. 8 (8), 467–477. 10.1038/nrclinonc.2011.76 21647195 PMC3423224

[B9] Crossland R. E. Norden J. Pearce K. F. Juric M. K. Lendrem C. Bibby L. A. (2016). Serum and extracellular vesicle micrornas MiR-423, MiR-199 and MiR-93*As biomarkers for acute graft versus host disease. Front. Immunol. 128 (22), 1446. 10.3389/fimmu.2017.01446 PMC5686047 29176973

[B10] Dewi I. S. Celik S. Karlsson A. Hollander Z. Lam K. McManus J. W. (2017). Exosomal miR-142-3p is increased during cardiac allograft rejection and augments vascular permeability through down-regulation of endothelial RAB11FIP2 expression. Cardiovasc. Res. 113 (5), 440–452. 10.1093/cvr/cvw244 28073833

[B11] Dogan N. Husing-Kabar A. Schmidt H. H. Cicinnati V. R. Beckebaum S. Kabar I. (2018). Acute allograft rejection in liver transplant recipients: Incidence, risk factors, treatment success, and impact on graft failure. J. Int. Med. Res. 46 (9), 3979–3990. 10.1177/0300060518785543 29996675 PMC6136012

[B12] Hamdorf M. Kawakita S. Everly M. (2017). The potential of MicroRNAs as novel biomarkers for transplant rejection. J. Immunol. Res. 2017, 4072364. 10.1155/2017/4072364 28191475 PMC5278203

[B13] Holzknecht Z. E. Platt J. L. (2000). The fine cytokine line between graft acceptance and rejection. Nat. Med. 6 (5), 497–498. 10.1038/74963 10802695

[B14] Israeli M. Klein T. Sredni B. Avitzur Y. Mor E. Bar-Nathen N. (2008). ImmuKnow: A new parameter in immune monitoring of pediatric liver transplantation recipients. Liver Transpl. 14 (6), 893–898. 10.1002/lt.21426 18508374

[B15] Jadlowiec C. C. Taner T. (2016). Liver transplantation: Current status and challenges. World J. Gastroenterol. 22 (18), 4438–4445. 10.3748/wjg.v22.i18.4438 27182155 PMC4858627

[B16] Kim W. R. Lake J. R. Smith J. M. Skeans M. A. Schladt D. P. Edwards E. B. (2016). Liver. Am. J. Transpl. 16 (2), 69–98. 10.1111/ajt.13668 26755264

[B17] Kohut T. J. Barandiaran J. F. Keating B. J. (2020). Genomics and liver transplantation: Genomic biomarkers for the diagnosis of acute cellular rejection. Liver Transpl. 26 (10), 1337–1350. 10.1002/lt.25812 32506790

[B18] Krenzien F. Keshi E. Splith K. Griesel S. Kamali K. Sauer I. M. (2019). Diagnostic biomarkers to diagnose acute allograft rejection after liver transplantation: Systematic review and meta-analysis of diagnostic accuracy studies. Front. Immunol. 10, 758. 10.3389/fimmu.2019.00758 31031758 PMC6470197

[B19] Kumar M. Ahmad T. Sharma A. Mabalirajan U. Kulshreshtha A. Agrawal A. (2011). Let-7 microRNA-mediated regulation of IL-13 and allergic airway inflammation. J. Allergy Clin. Immunol. 128 (5), 1077–1085. e1071-1010. 10.1016/j.jaci.2011.04.034 21616524

[B20] Kurashige J. Watanabe M. Iwatsuki M. Kinoshita K. Saito S. Hiyoshi Y. (2012). Overexpression of microRNA-223 regulates the ubiquitin ligase FBXW7 in oesophageal squamous cell carcinoma. Br. J. Cancer 106 (1), 182–188. 10.1038/bjc.2011.509 22108521 PMC3251856

[B21] Langmead B. Trapnell C. Pop M. Salzberg S. L. (2009). Ultrafast and memory-efficient alignment of short DNA sequences to the human genome. Genome Biol. 10 (3), R25. 10.1186/gb-2009-10-3-r25 19261174 PMC2690996

[B22] Ledeganck K. J. Gielis E. M. Abramowicz D. Stenvinkel P. Shiels P. G. VanCraenenbroeck A. H. (2019). MicroRNAs in AKI and kidney transplantation. Clin. J. Am. Soc. Nephrol. 14 (3), 454–468. 10.2215/CJN.08020718 30602462 PMC6419285

[B23] Levitsky J. Kandpal M. Guo K. Kleiboeker S. Sinha R. (2022). Donor-derived cell-free DNA levels predict graft injury in liver transplant recipients. Am. J. Transpl. 22 (2), 532–540. 10.1111/ajt.16835 34510731

[B24] Lin J. Li J. Huang B. Liu J. Chen X. Chen X. M. (2015). Exosomes: Novel biomarkers for clinical diagnosis. ScientificWorldJournal. 2015, 657086. 10.1155/2015/657086 25695100 PMC4322857

[B25] Lin Y. Wang L. Ge W. Hui Y. Zhou Z. Hu L. (2021). Multi-omics network characterization reveals novel microRNA biomarkers and mechanisms for diagnosis and subtyping of kidney transplant rejection. J. Transl. Med. 19 (1), 346. 10.1186/s12967-021-03025-8 34389032 PMC8361655

[B26] Lun A. Cho M. Y. Muller C. Staffa G. Bechstein W. O. Radke C. (2002). Diagnostic value of peripheral blood T-cell activation and soluble IL-2 receptor for acute rejection in liver transplantation. Clin. Chim. Acta. 320 (1-2), 69–78. 10.1016/s0009-8981(02)00045-1 11983203

[B27] Massoud O. Heimbach J. Viker K. Krishnan A. Poterucha J. Sanchez W. (2011). Noninvasive diagnosis of acute cellular rejection in liver transplant recipients: A proteomic signature validated by enzyme-linked immunosorbent assay. Liver Transpl. 17 (6), 723–732. 10.1002/lt.22266 21618694 PMC3293624

[B28] McLellan A. D. (2009). Exosome release by primary B cells. Crit. Rev. Immunol. 29 (3), 203–217. 10.1615/critrevimmunol.v29.i3.20 19538135

[B29] Meirelles Junior R. F. Salvalaggio P. Rezende M. B. Evangelista A. S. Guardia B. D. Matielo C. E. (2015). Liver transplantation: History, outcomes and perspectives. Einstein (Sao Paulo) 13 (1), 149–152. 10.1590/S1679-45082015RW3164 25993082 PMC4977591

[B30] Millan O. Sanchez-Fueyo A. Rimola A. Guillen D. Hidalgo S. Benitez C. (2009). Is the intracellular ATP concentration of cd4+t-cells a predictive biomarker of immune status in stable transplant recipients? Transplantation 88 (3), S78–S84. 10.1097/TP.0b013e3181afeba6 19667966

[B31] Mirzakhani M. Mohammadnia-Afrouzi M. Shahbazi M. Mirhosseini S. A. Hosseini H. M. Amani J. (2019). The exosome as a novel predictive/diagnostic biomarker of rejection in the field of transplantation. Clin. Immunol. 203, 134–141. 10.1016/j.clim.2019.04.010 31077803

[B32] Morita M. Chen J. J. Fujino M. Kitazawa Y. Sugioka A. Zhong L. (2014). Identification of microRNAs involved in acute rejection and spontaneous tolerance in murine hepatic allografts. Sci. Rep. 4, 6649. 10.1038/srep06649 25323448 PMC5377586

[B33] Naesens M. Anglicheau D. (2018). Precision transplant medicine: Biomarkers to the rescue. J. Am. Soc. Nephrol. 29 (1), 24–34. 10.1681/Asn.2017010004 28993504 PMC5748900

[B34] Obregon C. Rothen-Rutishauser B. Gitahi S. K. Gehr P. Nicod L. P. (2006). Exovesicles from human activated dendritic cells fuse with resting dendritic cells, allowing them to present alloantigens. Am. J. Pathol. 169 (6), 2127–2136. 10.2353/ajpath.2006.060453 17148675 PMC1762484

[B35] Perottino G. Harrington C. Levitsky J. (2022). Biomarkers of rejection in liver transplantation. Curr. Opin. Organ Transpl. 27 (2), 154–158. 10.1097/MOT.0000000000000959 35232928

[B36] Rastogi A. (2022). Liver transplant biopsy interpretation: Diagnostic considerations and conundrums. Indian J. Pathol. Microbiol. 65 (2), 245–257. 10.4103/ijpm.ijpm_1090_21 35435355

[B37] Schütz E. Fischer A. Beck J. Harden M. Koch M. Wuensch T. (2017). Graft-derived cell-free DNA, a noninvasive early rejection and graft damage marker in liver transplantation: A prospective, observational, multicenter cohort study. PLoS Med. 14 (4), e1002286. 10.1371/journal.pmed.1002286 28441386 PMC5404754

[B38] Shenoy K. V. Solomides C. Cordova F. Rogers T. J. Ciccolella D. Criner G. J. (2012). Low CD4/CD8 ratio in bronchus-associated lymphoid tissue is associated with lung allograft rejection. J. Transpl. 2012, 928081. 10.1155/2012/928081 PMC3423936 22928088

[B39] Thery C. Amigorena S. Raposo G. Clayton A. (2006). Isolation and characterization of exosomes from cell culture supernatants and biological fluids. Curr. Protoc. Cell Biol. 3, Unit 3.22. 10.1002/0471143030.cb0322s30 18228490

[B40] Thery C. (2011). Exosomes: Secreted vesicles and intercellular communications. F1000 Biol. Rep. 3, 15. 10.3410/B3-15 21876726 PMC3155154

[B41] Valadi H. Ekstrom K. Bossios A. Sjostrand M. Lee J. J. Lotvall J. O. (2007). Exosome-mediated transfer of mRNAs and microRNAs is a novel mechanism of genetic exchange between cells. Nat. Cell Biol. 9 (6), 654–659. 10.1038/ncb1596 17486113

[B42] van der Vlist E. J. Arkesteijn G. J. van de Lest C. H. Stoorvogel W. Nolte-'t Hoen E. N. Wauben M. H. (2012). CD4(+) T cell activation promotes the differential release of distinct populations of nanosized vesicles. J. Extracell. Vesicles 1, 18364. 10.3402/jev.v1i0.18364 PMC3760647 24009884

[B43] Wang J. P. Li X. Wu X. Q. Wang Z. W. Zhang C. Cao G. H. (2019). Expression profiling of exosomal mirnas derived from the peripheral blood of kidney recipients with dgf using high-throughput sequencing. Biomed. Res. Int. 2019, 1759697. 10.1155/2019/1759697 31309102 PMC6594342

