# Supplementary material for: Serum extracellular vesicle MicroRNAs as candidate biomarkers for acute rejection in patients subjected to liver transplant
Source: Front Genet. 2022 Oct 13;13:1015049. doi: 10.3389/fgene.2022.1015049 (PMC9606588; doi:10.3389/fgene.2022.1015049)
Supplement: Supplementary file 1 [file Table1.DOCX]

Supplementary Material

**Table S1 Characteristics of AR and non-AR Patients**

| Variable | AR Patients  (n = 30) | non-AR Patients (n = 30) | P Value |
| --- | --- | --- | --- |
| Age, y | 53.4 ± 8.4 | 49.2 ± 7.6 | NS |
| Sex, male/female | 24/6 | 20/10 | NS |
| Body mass index, kg/m^2^ | 21.7 ± 3.3 | 19.2 ± 4.5 | NS |
| Etiology  Liver cirrhosis  Hepatitis B virus related  Hepatitis C virus related  Alcohol related  Other  HCC | 25  17  3  1  4  5 | 26  17  3  1  5  4 | NS |
| Child-Pugh score  B  C | 12  18 | 14  16 | NS |
| MELD score | 19.2 ± 2.2 | 20.8 ± 4.3 | NS |
| ABO compatible  Yes  No | 29  1 | 30  0 | NS |
| Cold ischemia time, h | 7.8 ± 1.8 | 6.9 ± 2.5 | NS |
| Warm ischemia time, min | 14.9 ± 8.7 | 12.6 ± 3.8 | NS |
| Anhepatic phase, min | 46.8 ± 8.4 | 47.2 ± 9.6 | NS |
| Immunosuppressive drugs  Tacrolimus+ MMF+ steroids  Cyclosporine+ MMF+ steroids | 18  12 | 21  9 | <0.05 |
| Mean immunosuppression concentration in the first week  Low  Normal  High | 16  14  0 | 4  14  12 | <0.05 |
| Time until rejection onset, days post-LT | 34.8 ± 28.9 | --- | --- |
| Time to biopsy, days post-LT | 43.3 ± 30.7 | --- | --- |
| Banff RAI | 7.3 ± 1.7 | --- | --- |
| Prognosis rate, % | 90 | 100 | NS |

*Abbreviations:* MELD, Model for End-Stage Liver Disease; MMF, Mycophenolate Mofetil; RAI, Rejection Activity Index; NS, not significant

**Table S2 Primer Sequences Used for qRT-PCR in This Study.**

| Gene | Sequence (5’-3’) |
| --- | --- |
| miRNA-F | ATCCAGTGCGTGTCGTG |
| miRNA-152-3p-RT | GTCGTATCCAGTGCGTGTCGTGGAGTCGGCAATTGCACTGGATACGACCCAAGTT |
| miRNA-152-3p-R | TGCTTCAGTGCATGACA |
| miRNA-148-3p-RT | GTCGTATCCAGTGCGTGTCGTGGAGTCGGCAATTGCACTGGATACGACACAAAGT |
| miRNA-148-3p-R | TGCTTCAGTGCACTACAGA |
| let-7e-5p-RT | GTCGTATCCAGTGCGTGTCGTGGAGTCGGCAATTGCACTGGATACGACAACTATA |
| let-7e-5p-R | ATCCAGTGCGTGTCGTG |
| miRNA-486-3p-RT | GTCGTATCCAGTGCGTGTCGTGGAGTCGGCAATTGCACTGGATACGACATCCTGT |
| miRNA-486-3p-R | TGCTCGGGGCAGCTCAGT |
| miRNA-199a-3p-RT | GTCGTATCCAGTGCGTGTCGTGGAGTCGGCAATTGCACTGGATACGACTAACCAA |
| miRNA-199a-3p-R | TGCTACAGTAGTCTGCACA |
| miRNA-340-5p-RT | GTCGTATCCAGTGCGTGTCGTGGAGTCGGCAATTGCACTGGATACGACAATCAGT |
| miRNA-340-5p-R | TGCTTTATAAAGCAATGAG |
| miRNA-142-3p-RT | GTCGTATCCAGTGCGTGTCGTGGAGTCGGCAATTGCACTGGATACGACTCCATAA |
| miRNA-142-3p-R | TGCTTGTAGTGTTTCCTACT |
| miRNA-15b-5p-RT | GTCGTATCCAGTGCGTGTCGTGGAGTCGGCAATTGCACTGGATACGACTGTAAAC |
| miRNA-15b-5p-R | TGCTTAGCAGCACATCATG |
| miRNA-215-5p-RT | GTCGTATCCAGTGCGTGTCGTGGAGTCGGCAATTGCACTGGATACGACGTCTGTC |
| miRNA-215-5p-R | TGCTATGACCTATGAATT |
| miRNA-192-5p-RT | GTCGTATCCAGTGCGTGTCGTGGAGTCGGCAATTGCACTGGATACGACGGCTGTC |
| miRNA-192-5p-R | TGCTCTGACCTATGAATT |
| miRNA-223-3p-RT | GTCGTATCCAGTGCGTGTCGTGGAGTCGGCAATTGCACTGGATACGACTGGGGTA |
| miRNA-223-3p-R | ATCCAGTGCGTGTCGTG |
| miRNA-106b-RT | GTCGTATCCAGTGCGTGTCGTGGAGTCGGCAATTGCACTGGATACGACATCTGCA |
| miRNA-106b-R | TGCTTAAAGTGCTGACAG |
| U6-RT | CGCTTCACGAATTTGCGTGTCAT |
| U6-F | GCTTCGGCAGCACATATACTAAAAT |
| U6-R | CGCTTCACGAATTTGCGTGTCAT |

**Table S3 small RNA category.**

| Types | non-AR | non-AR (percent) | AR | AR (percent) |
| --- | --- | --- | --- | --- |
| total | 11982753 | 100.00% | 13990228 | 100.00% |
| rRNA | 3970480 | 33.13% | 5295654 | 37.85% |
| tRNA | 493328 | 4.12% | 614382 | 4.39% |
| scRNA | 0 | 0.00% | 0 | 0.00% |
| snRNA | 300 | 0.00% | 261 | 0.00% |
| snoRNA | 12562 | 0.10% | 5867 | 0.04% |
| Repbase | 571796 | 4.77% | 2205204 | 15.76% |
| Unannotated^*^ | 6934287 | 57.88% | 5868860 | 41.96% |

^*^ miRNA included

**Table S4 Quantification of miRNA expression level by TPM (Parts of data).**

| miRNA | non-AR.readcount | AR.readcount | non-AR.tpm | AR.tpm |
| --- | --- | --- | --- | --- |
| let-7a-5p | 3488 | 6120 | 922.58340 | 7782.74443 |
| let-7b-5p | 8080 | 14495 | 2137.17715 | 18433.1504 |
| miR-100-5p | 14449 | 3673 | 3821.79117 | 4670.91835 |
| miR-10a-5p | 31699 | 973 | 8384.45278 | 1237.35463 |
| miR-125a-5p | 378 | 51 | 99.98180 | 64.85620 |
| miR-1290 | 869 | 4020 | 229.85234 | 5112.19487 |
| miR-145-3p | 508 | 12 | 134.36707 | 15.26028 |
| miR-146a-5p | 4253 | 561 | 1124.92753 | 713.41824 |
| miR-148a-3p | 1717190 | 58722 | 454200.4 | 74676.1959 |
| miR-152-3p | 3161 | 243 | 836.09121 | 309.02073 |
| miR-184-5p | 1757 | 3146 | 464.73022 | 4000.73758 |
| miR-192-5p | 63450 | 7346 | 16782.6597 | 9341.8367 |
| miR-194-5p | 2925 | 173 | 773.66870 | 220.00241 |
| miR-199a-3p | 2145 | 106 | 567.35705 | 134.79916 |
| miR-200a-3p | 602 | 29 | 159.23027 | 36.87901 |
| miR-203a-3p | 140 | 306 | 37.03029 | 389.13722 |
| miR-215-5p | 658 | 119 | 174.04239 | 151.33114 |
| miR-24-3p | 3519 | 521 | 930.78296 | 662.55062 |
| miR-30a-5p | 5239 | 381 | 1385.72662 | 484.51399 |
| miR-328-3p | 599 | 22 | 158.43677 | 27.97718 |
| miR-372-3p | 1842 | 90 | 487.21290 | 114.45212 |
| miR-375-3p | 2209 | 171 | 584.28518 | 217.45903 |
| miR-423-5p | 11394 | 37975 | 3013.73718 | 48292.4379 |
| miR-99a-5p | 137884 | 4702 | 36470.6106 | 5979.48764 |

**Table S5 Differential expressed miRNA between AR and non-AR patients. (Parts of data)**

| miRNA | non-AR.readcount | AR.readcount | | Log_2_Foldchange | P value (adj.) |
| --- | --- | --- | --- | --- | --- |
| let-7d-5p | 405 | | 705 | 2.1774 | 0.00443 |
| let-7e-5p | 61 | | 150 | 1.9919 | 0.01615 |
| miR-1-3p | 3027 | | 5268 | 1.9554 | 0.01001 |
| miR-125b-5p | 606 | | 67 | -2.1033 | 0.00470 |
| miR-130a-3p | 283 | | 27 | -2.35735 | 0.00252 |
| miR-16-5p | 161 | | 225 | 2.16987 | 0.00993 |
| miR-193a-5p | 1702 | | 182 | -2.82883 | 0.00122 |
| miR-199a-3p | 2145 | | 106 | -3.54877 | 0.00000 |
| miR-27b-3p | 38610 | | 4865 | -2.24045 | 0.00418 |
| miR-223 | 49 | | 99 | 1.97388 | 0.02397 |
| miR-28a-3p | 3016 | | 333 | -2.46882 | 0.00087 |
| miR-29a-3p | 1119 | | 148 | -2.14205 | 0.00339 |
| miR-30d-5p | 14535 | | 2281 | -1.79663 | 0.01199 |
| miR-340-5p | 692 | | 105 | -1.99935 | 0.00872 |
| miR-378c | 1176 | | 104 | -2.44424 | 0.00112 |
| miR-450b-5p | 271 | | 47 | -1.69166 | 0.03855 |
| miR-623 | 985 | | 49 | -1.71823 | 0.04983 |
| miR-653 | 23 | | 379 | 4.20503 | 0.00142 |

**Table S6 Go enrichment analysis results. (Parts of data)**

| GO_accession | Description | Term_type | p Value |
| --- | --- | --- | --- |
| GO:0043231 | intracellular membrane-bounded organelle | cellular_component | 2.91E-05 |
| GO:0006366 | transcription by RNA polymerase II | biological_process | 2.90E-05 |
| GO:0043227 | membrane-bounded organelle | cellular_component | 0.00078 |
| GO:0005654 | nucleoplasm | cellular_component | 4.45E-05 |
| GO:1901362 | organic cyclic compound biosynthetic process | biological_process | 4.15E-05 |
| GO:0019438 | aromatic compound biosynthetic process | biological_process | 7.94E-05 |
| GO:0009891 | positive regulation of biosynthetic process | biological_process | 0.00021 |
| GO:0031328 | positive regulation of cellular biosynthetic process | biological_process | 0.00026 |
| GO:0005667 | transcription regulator complex | cellular_component | 0.00115 |
| GO:0043229 | intracellular organelle | cellular_component | 0.00158 |
| GO:0032774 | RNA biosynthetic process | biological_process | 0.00038 |
| GO:0003700 | DNA-binding transcription factor activity | molecular_function | 6.36E-08 |
| GO:0005524 | ATP binding | molecular_function | 2.34E-06 |
| GO:0097659 | nucleic acid-templated transcription | biological_process | 0.00040 |
| GO:0015629 | actin cytoskeleton | cellular_component | 0.00205 |
| GO:0031326 | regulation of cellular biosynthetic process | biological_process | 0.00046 |
| GO:0035556 | intracellular signal transduction | biological_process | 0.00139 |
| GO:0051252 | regulation of RNA metabolic process | biological_process | 0.00161 |
| GO:0140110 | transcription regulator activity | molecular_function | 6.38E-05 |
| GO:0003682 | chromatin binding | molecular_function | 0.00036 |
| GO:0008104 | protein localization | biological_process | 0.00178 |
| GO:0005829 | cytosol | cellular_component | 0.00334 |
| GO:0007155 | cell adhesion | biological_process | 0.00239 |

**Table S7 KEGG pathway terms (Top 20)**

| pathway_term | rich_factor | p value | gene_number |
| --- | --- | --- | --- |
| Protein processing in endoplasmic reticulum | 1.07 | 0.00039 | 111 |
| Axon guidance | 1.07 | 0.00268 | 84 |
| MicroRNAs in cancer | 1.07 | 0.00472 | 76 |
| Human papillomavirus infection | 1.07 | 0.00672 | 71 |
| Choline metabolism in cancer | 1.07 | 0.00892 | 67 |
| Cell adhesion molecules | 1.07 | 0.01103 | 64 |
| Gastric cancer | 1.05 | 0.0113 | 116 |
| Proteoglycans in cancer | 1.07 | 0.01184 | 63 |
| Pathways in cancer | 1.06 | 0.02166 | 81 |
| PD-L1 expression and PD-1 checkpoint pathway in cancer | 1.07 | 0.02235 | 54 |
| Signaling pathways regulating pluripotency of stem cells | 1.07 | 0.02763 | 51 |
| Lysosome | 1.04 | 0.04251 | 112 |
| Tuberculosis | 1.07 | 0.04527 | 44 |
| Regulation of actin cytoskeleton | 1.07 | 0.06002 | 40 |
| Spinocerebellar ataxia | 1.07 | 0.07956 | 36 |
| Morphine addiction | 1.07 | 0.07956 | 36 |
| Nicotine addiction | 1.07 | 0.0916 | 34 |
| Glycosaminoglycan biosynthesis - heparan sulfate / heparin | 1.07 | 0.09829 | 33 |
| Ubiquitin mediated proteolysis | 1.04 | 0.10326 | 74 |
| Glycerophospholipid metabolism | 1.07 | 0.10546 | 32 |

**
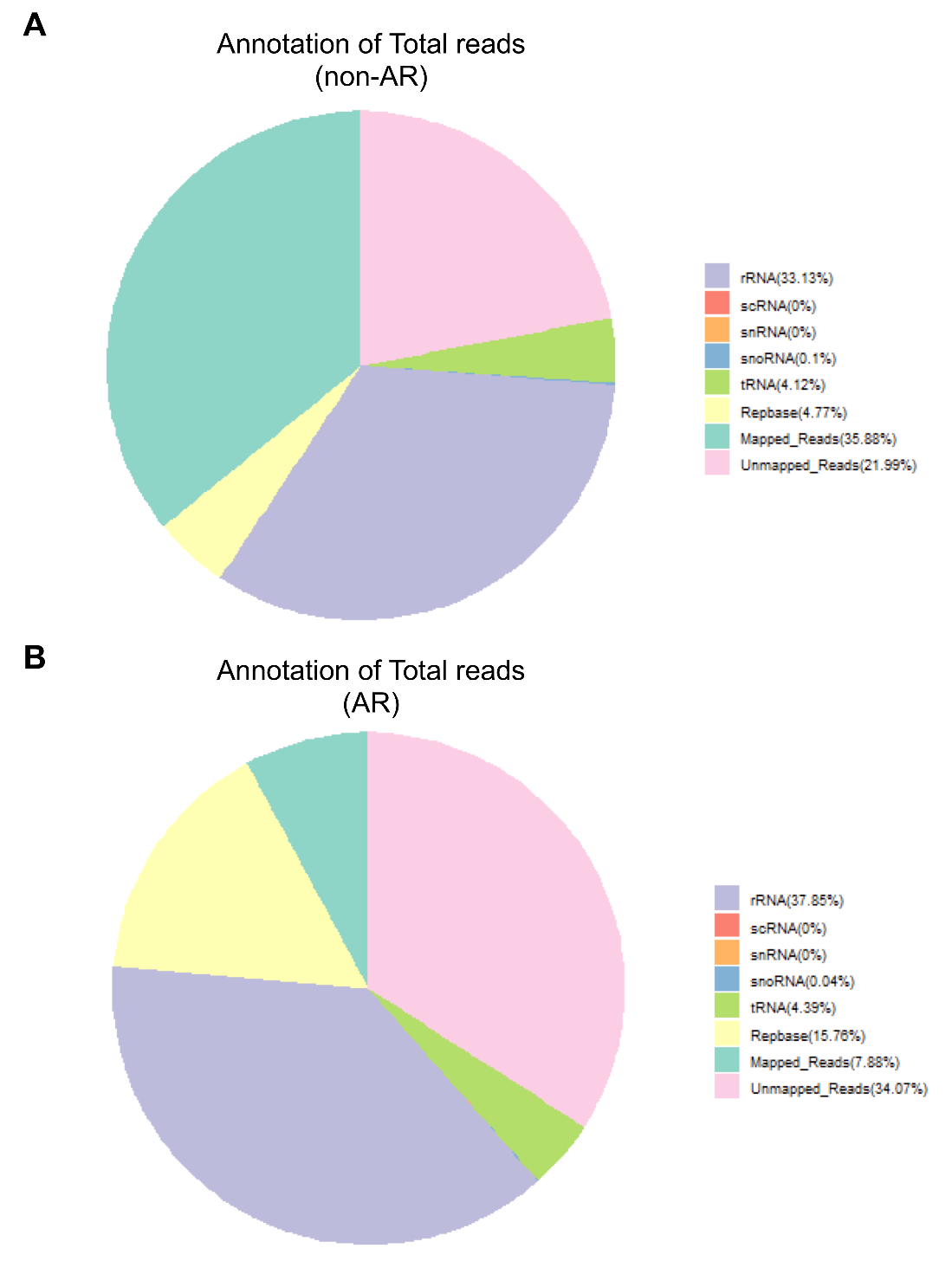
**

**Figure S1** **The sRNA classification annotation statistics of the clean reads.** (A) non-AR patients. (B) AR patients.


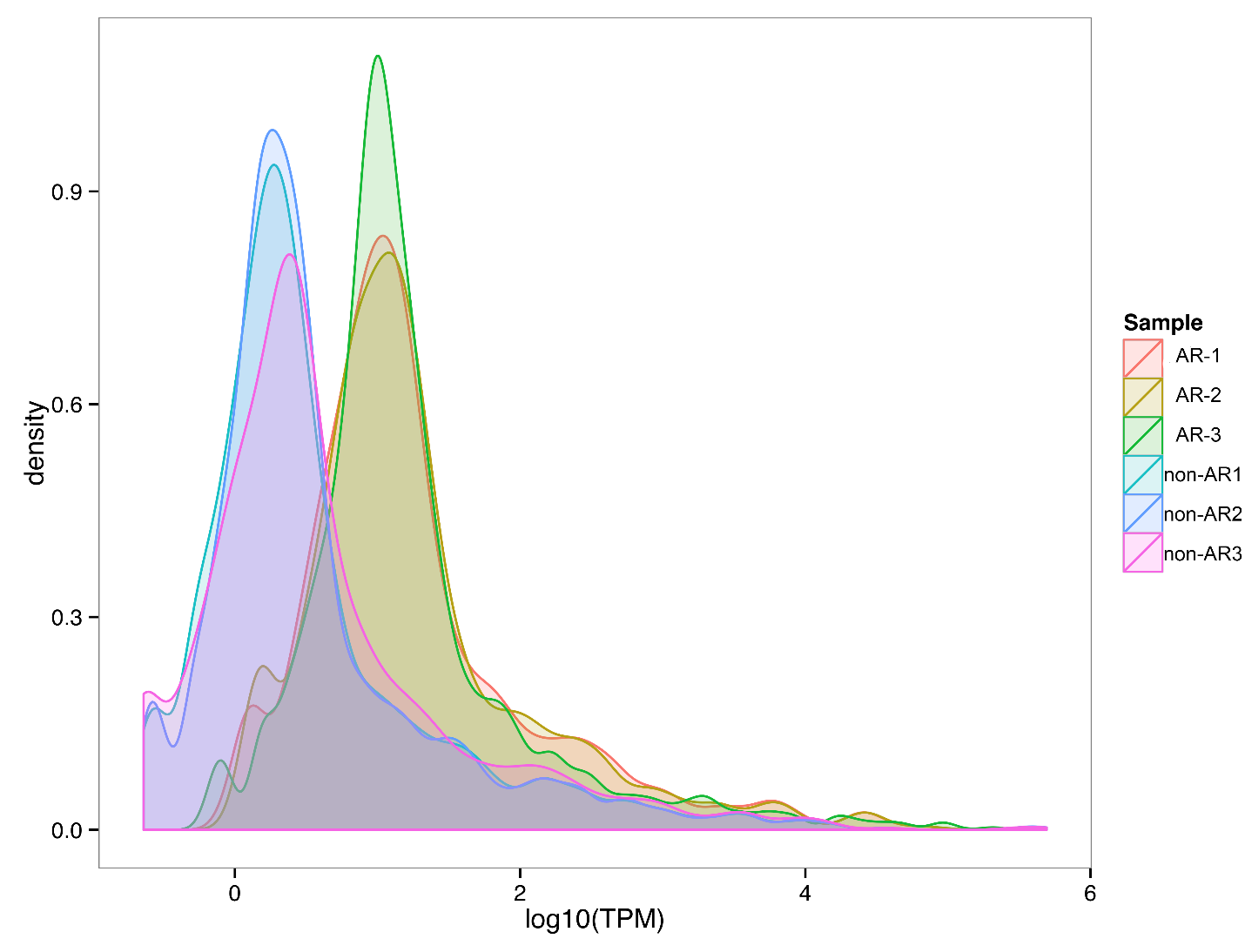


**Figure S2** **The density distribution of transcript per million (TPM)**. Horizontal axis: Value of miRNA’s log10 (TPM). Vertical axis: the density of matched log10 (TPM).


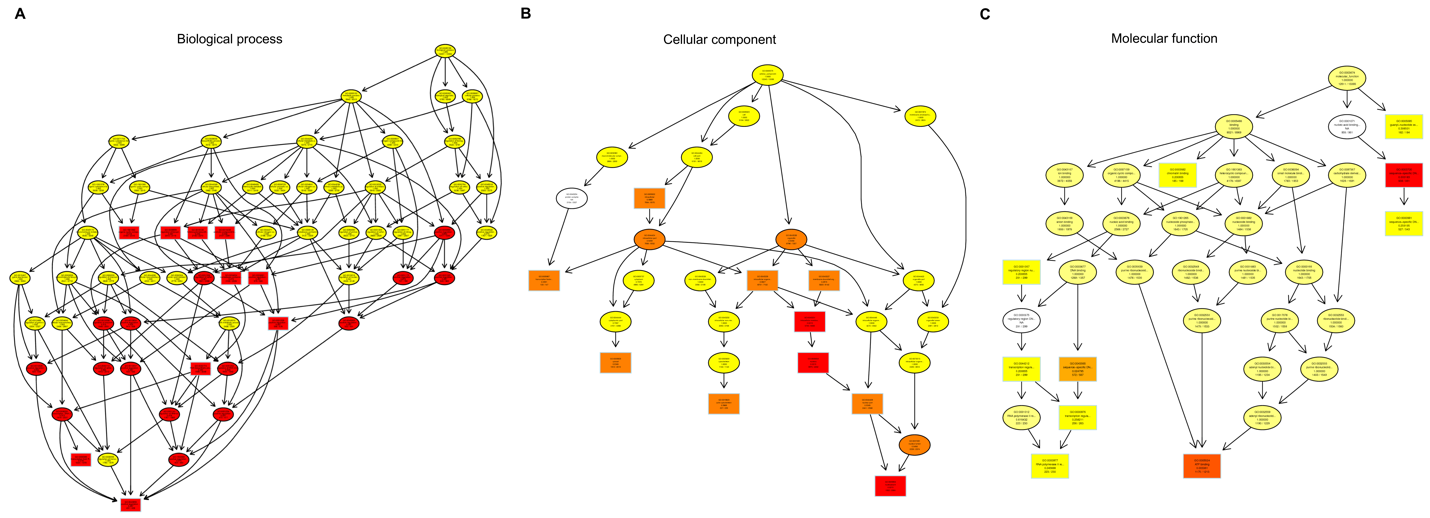


**Figure S3** GO function classification target genes of known differential expressed miRNAs in AR patients. From left to right they are biological process (A), cellular component (B), and molecular function (C).
